# Emotional burnout in psychiatrists during the war: experience of Ukraine

**DOI:** 10.1192/j.eurpsy.2024.1736

**Published:** 2024-08-27

**Authors:** N. O. Maruta, V. D. Mishiev, T. I. Levin, H. A. Prib, A. R. Markov

**Affiliations:** 1 ”Institute of Neurology, Psychiatry and Narcology of the NAMS of Ukraine” SI, Kharkiv; 2 P.L. Shupyk National University of the Health Care of Ukraine; 3 Academy of Labour, Social Relations and Tourism, Kyiv, Ukraine

## Abstract

**Introduction:**

Psychiatrists being one of the significant groups associated with one of the highest risks of emotional burnout (EB). The risks of EB increase significantly in the conditions of war, which places increased demands on their physical, mental and psychological resources, and determines the relevance and necessity of studying the predictors, clinical phenomenology, psychological and psychopathological mechanisms of EB, and necessitates the development of innovative approaches to its corrections.

**Objectives:**

The study the features of EB among psychiatrists in war period.

**Methods:**

The examination included the usage of clinical-psychopathological, psychodiagnostic and psychometric research methods.

**Results:**

The study sample consisted of 120 psychiatrists who worked in Kyiv in the period from February 24, 2022, during 2022. 69.2 of psychiatrists working in Kyiv during the war have manifestations of EB of varying intensity and clinical variability. All of them have signs of professional maladaptation (PM), deterioration of well-being, somatovegetative and dyssomnia disorders, deformation of social ties and decrease in motivation to work. In 47.5%, the formation of symptoms of tension, resistance and exhaustion are observed, 21.7% have clinically complited and formed all manifestations of EB and PM.

The leading diagnostic and prognostic marker of EB is PM, the manifestations of which are the first consequence of the imbalance of the processes of performing professional duties and internal resources, which will ensure their optimal implementation.

A mathematical model of the development and forecast of PM, as a leading descriptor of EB, was developed, which considers the state of socio-demographic characteristics (age, work experience, and the total quality of life indicator), affective indicators (objective and subjective manifestations of depression and anxiety) and psychosocial features (social-psychological adaptation). The use of this model makes it possible to determine 4 risk groups for the development of PM (low, moderate, high, very high), based on which personalized approaches to the diagnosis, therapy and prevention of EB among psychiatrists during the war have been developed.

**Conclusions:**

The implementation and further evaluation of these approaches proved their effectiveness in eliminating the manifestations of EB and PM, normalizing the mental state with the levelling of psychopathological symptoms, improving the socio-psychological adaptation and quality of life of psychiatrists.

**Disclosure of Interest:**

None Declared

